# Elution profile of cationic and anionic adsorbate from exhausted adsorbent using solvent desorption

**DOI:** 10.1038/s41598-022-05805-8

**Published:** 2022-01-31

**Authors:** Himanshu Patel

**Affiliations:** Department of Applied Science and Humanities, Pacific School of Engineering, Kadodara Palsana Road (NH-8), V: Sanki, Ta. Palsana, Surat, Gujarat 394305 India

**Keywords:** Chemical engineering, Environmental impact

## Abstract

Present invention involves to study the elution profile of anionic and cationic compounds from exhausted adsorbents using various eluents. Batch elution studies of anionic components like Congo Red dye and Carbonate ion; and cationic compounds such as Methylene blue dye and Cadmium metal from previously used naturally prepared adsorbents i.e. Gulmohar (*Delonix regia*) leaf powder—GLP; and Neem (*Azadirachta indica*) leaf powder—NLP and their derivatives were conducted. Different eluents used for batch study were various acids and alkaline solution having various concentration and solvents having different functional groups in seven sorption–desorption cycles. The batch data were accessed by kinetic models (Pseudo First-, Pseudo Second-order, Intra-particle and Elovic equation). Column elution experiments of Congo red and Cadmium from NLP and activated charcoal from NLP (AC-NLP) respectively was performed using selected eluent. Sorption and elution process plots and parameters and life cycle plots for seven sorption–desorption cycles were evaluated and discussed. From desorption efficiencies, it revealed that desorption exploration is predominately depends upon pH factor.

## Introduction

In the World, industries are want to develop in such way, the concept of sustainable development would be maintained. But, they have several difficulties including contaminant effluent, air emission cease, solid waste recycle, noise entrapment, etc. Out of these, mostly complication of solid waste management are facing by industrial personal^1^. It is estimated that industrial solid waste generation in some regions is 1900 million tonnes per annum and this quantity will be double in below 20 years. This industrial solid waste was facing challenges of collection, processing and disposal^2^. Solid waste are preferably disposal through landfill, incineration and composting. In landfilling, solid waste leachate would be created land pollution. Also, it also liberates methane gas, which is first greenhouse gas. Further, incineration of solid waste is responsible for generating highly hazardous air pollutants such as carbon oxides (COx), nitrogen oxides (NOx), sulphur oxides (SOx), polyaromatic hydrocarbons, and heavy metals. Composting of solid waste required large area and proper environmental conditions^3^. World-wide, adsorption process is widely utilized for water and wastewater treatment for eliminating different contaminations. In industries, the adsorption is used as a tertiary treatment in effluent treatment plant (ETP), in which activated charcoal as an adsorbent is used. Also, bed of silica sand, anthracite coal or garnet sand or mixture of combination of these materials are also used as tertiary filtration^4^. Also, adsorption process is used for purification, isolation, catalyzation, dehumidification, fermentation, odour removal, hydrocarbon fractionation, etc. In adsorption process, adsorption capacity of activated charcoal, coal or sand is completed and considered as exhausted adsorbent after some-time. It is thereafter recognized as solid hazardous waste and disposed through landfilling^5,6^. Beside the char, natural materials like plant root, leaf and agri-waste are easily available, cheap, retain sustainable development, safe to use, substitution to traditional adsorbents but adsorption capacity is comparatively low. For proper solid waste management and adoption of sustainable development, several scientists are being worked for regenerating the exhausted adsorbent and re-utilized. Different techniques such as thermal regeneration, supercritical fluid extraction, biological, advance oxidation, micro-wave, ultra-sound, ozonation, desorption, etc. are available for regeneration of used adsorbent. Each method have their merits and de-merits^7,8^. Desorption i.e. reserve process of adsorption is superior method than other regeneration methods. Further, desorption study clearly justify the mechanism of adsorption i.e. physical or chemical adsorption and also, give the information about the stability of the adsorbent for further use^9^. There are two types of desorption. Thermal desorption is effective, but it is expensive, release inadequate pollutants and require pre-process, while solvent desorption is referred as simple yet effective, less time consuming, eco-friendly and cheap method. In solvent desorption, exhausted adsorbent is treated with liquid materials, called eluent, like acid, alkali and organic solvent through batch or column experiments. Selection of eluent in such way that exhausted adsorbent is liberated the adsorbate. After desorption, exhausted adsorbent is regenerated and re-used^10^.

Present paper is described the feasibility of desorption study for metal- and dye-exhausted adsorbent using different eluents such as acid, alkali and solvent. Comparative studies of eluents and type of solvent desorption (batch and column) were evaluated with respective to desorption capacity. Data of batch desorption is evaluated with kinetic isotherm. Column desorption was accomplished by sorption and elution process plots and parameters; and activity-indicator equations.

## Methods and materials

For all batch experiments, five acids (hydrochloric acid, sulphuric acid, nitric acid, acetic acid, and ethylene diamine tetraacetic acid—EDTA), five alkalis (sodium hydroxide, potassium hydroxide, lithium hydroxide, calcium hydroxide and sodium bicarbonate); and five organic solvents (rectified spirit, acetone, acetaldehyde, toluene and dichloromethane—DCM) as an eluent were utilized. Different concentrations i.e. 0.05, 0.10, 0.15 and 0.20 M solutions of acid and alkali was prepared using distilled water. Selection of these eluents is due to the fact that experiment covers entire range of strong as well as weak acid and alkaline solution; and also, solvent having different functional groups like alcohol, ketone, aldehyde, hydrocarbon and halogen. Also, elution of adsorbate is selected that anionic as well as cationic material is used in this experiment. Batch and column desorption studies were conducted as per Table 1. In Table 1, Gulmohar (*Delonix regia*) leaf powder—GLP, Neem (*Azadirachta indica*) leaf powder—NLP, NLP, NLP and activated charcoal from NLP (AC-NLP) were used for elution of Congo Red dye, Carbonate ion, Methylene Blue dye, Cadmium and Cadmium metal respectively for batch desorption. Previously, these adsorbents were exhausted with different adsorbent dose, initial concentration and temperature as per Table 1. Batch desorption study was conducted by taking 1.0 g exhausted adsorbent and adding 250.0 mL eluent for contact duration of 6 h and temperature of 300 K with occasion stirring of 2 min with time elapse of every 1 h. Thereafter, solution was filtered and quantity of adsorbate was determined from filtrate. Here, optimum contact duration was 6 h for batch desorption study. For chemical kinetic isotherm, batch desorption study was performed for contact duration of 30, 60, 120, 180, 240 and 300 min maintaining another batch parameters. Four chemical kinetic isotherms i.e. Pseudo-First order, Pseudo-Second order, Intra-particle diffusion and Elovich isotherm were used to understand desorption pathways and rate. Respective graphs of isotherms were plotted and their correlation coefficient values were determined^11^.

**Table 1:** Sample details used for desorption study.

Batch study					Adsorbed amount of adsorbate (% adsorption)	References
Adsorbent	Adsorbate	Previously used parameter for adsorption				
		Adsorbent dose (g/L)	Initial conc. (mg/L)	Temperature (K)		
Gulmohar (*Delonix regia*) leaf powder—GLP	Congo Red dye	12.0	60	370	51 mg/L (85%)	Patel^20^
Neem (*Azadirachta indica*) leaf powder—NLP	Carbonate ion	13.5	100	315	50 mg/L (50%)	–
NLP	Methylene Blue dye	3.0	200	300	166 mg/L (83%)	Patel and Vashi^21^
NLP	Cadmium (Cd)	13.5	100	315	50 mg/L (50%)	–
Activated charcoal from NLP (AC-NLP)	Cadmium (Cd)	10.0	100	315	50 mg/L (50%)	Patel ^12^

Column study					Adsorbed amount of adsorbate (% adsorption)	References
Adsorbent	Adsorbate	Previously used parameter for adsorption				
		Bed height (cm)	Initial conc. (mg/L)	Flow rate (mL/min)		
GLP	Congo Red dye	10	60	10	60 mg/L (100%)	Patel^20^
Activated charcoal from NLP (AC-NLP)	Cadmium (Cd)	10	25	10	25 mg/L (100%)	Patel^12^

Congo red dye exhausted GLP and Cadmium exhausted AC-NLP were undergone column desorption study using selected eluents to compare the batch and column desorption study. Previously used column for adsorption was utilized in desorption study^12^. Choice of Cadmium metal among another five metals i.e. Copper(II), Chromium(II), Zinc(II), Nickel(II) and Lead(II) is due to the fact that it was least removal metal during previous batch as well as column adsorption experiment. It is estimated that if exhausted Cadmium would be desorbed, then another metals would be easily desorbed. Earlier 100% Congo red dye was adsorbed onto GLP using bed height, initial dye concentration and flow rate of 10 cm, 60 mg/L and 10 mL/min. Also, 25 mg/L cadmium was adsorbed onto AC-NLP using bed height of 10 cm, initial concentration 25 mg/L and flow rate of 10 mL/min as per Table 1. In column desorption experiments, each eluent was passed through 10 cm column with defined flow rates and, elution amount of adsorbate was estimated from eluent. Breakthrough desorption and elution curve were plotted to analyse elution profile. Various column sorption and elution process parameters for seven sorption–desorption cycles such as uptake capacity (Q), breakthrough time (t_b_), exhaustion time (t_e_), mass transfer zone (∆t), bed height (Z), critical bed length (Z_m_) i.e. bed length required to obtain the breakthrough time t_b_ at t = 0, elution time, rate constant (dC/dt for 1 h), percentage removal and elution efficiency for Congo red and cadmium were calculated^13^. Also, life of column was determined using activity-indicator equations ^14^, which are follows.

$$\begin{aligned} {\text{t}}_{{\text{b}}} & = {\text{ t}}_{{{\text{b,}}0}} + {\text{ kt}}_{{\text{b}}} x \\ {\text{Q }} & = {\text{ Q}}_{0} + {\text{ k}}_{{\text{Q}}} x \\ {\text{Z}}_{{\text{m}}} & = {\text{ Z}}_{{{\text{m}},0}} + {\text{ kZ}}_{{\text{m}}} x \\ \end{aligned}$$

where, *x* is number of cycle, t_b,0_, Q_0_, and Z_m,0_ are the initial breakthrough time, column uptake, and critical bed length, respectively. Also, kt_b_, k_Q_, and kZ_m_ represent life factors corresponding to the breakthrough time, uptake, and critical bed length, respectively. All these life factors were determined by plotting the graphs of breakthrough time, uptake capacity and critical bed length vs*.* number of cycle.

Here, most of exhausted adsorbents were taken from previous experiments to maintain sustainability. In batch as well as column desorption study, first exhausted adsorbent was washed with water thrice and dried at 50–60 °C for 48 h. Thereafter, each exhausted adsorbent was stored under glass bottle and then, vacuum desiccator to preserve its integrity. Also, Table 1 represents the batch and column parameters by which these adsorbents were exhausted earlier. Also, it mentioned the adsorbed amount of adsorbate in previous experiments. Thereafter, each adsorbent was further utilized for same adsorption study (batch or column) of similar adsorbate. After completion of one cycle i.e. adsorption (generating spent adsorbent) and desorption (re-generating adsorbent from spent adsorbent), desorption efficiency (%) were determined using following equation. Numbers of cycles were run to analyse the feasibility of desorption study.

$$Desorption \;efficiency \;(\% ) = \frac{{C_{des} }}{{C_{ads} }} \times 100$$

where, C_des_ is the concentration of adsorbate desorbed in mg/g and C_ads_ is the concentration of adsorbate adsorbed in mg/g^15^.

All the materials were purchased from Sigma-Aldrich. For determination of acidic dye, basic dye and metal, double beam UV–Visible Spectrophotometer (ELICO SL Double Beam UV–VIS Spectrophotometer) was used. Quantity of Congo red and Methylene Blue dye was estimated using at λ_max_ = 500 and 665 nm respectively. Also, Cadmium and carbonate ion was determined was analysed as per Standard Methods for Examination of Water and Wastewater^16^. Cadmium was analysed in Flame Atomic Absorption Spectroscopy (AAS) (Model: PinAAcle 900, Pelkin Elmer). All the experiments were conducted three times and average values were taken.

## Results and discussion

### Batch study

Batch desorption study was conducted using different types of adsorbates, adsorbents and eluents, in which seven cycles of adsorption-desertion were performed. Table 2 represents the experimental results of Congo red dye and Carbonate ion elution from exhausted GLP and NLP respectively using acids, alkali and solvents as an eluent. Using 0.05–0.20 M acids (hydrochloric acid, sulphuric acid, nitric acid, acetic acid and EDTA), least desorption efficiencies of Congo red dye from GLP were achieved to 1.0–2.3% after first cycles. Also, desorption efficiencies were attained up to 15.1% using all the solvents (rectified spirit, acetone, acetaldehyde, toluene and DCM) as an eluent after first cycles, but, higher desorption efficiencies were attained to 87.5, 98.5, 80.2 and 74.5% using 0.05, 0.10, 0.15 and 0.20 M sodium hydroxide solution respectively using first cycle. Thereafter, efficiencies were continuously decreased by consecutive cycles, so, efficiencies were decreased by 50.4, 58.4, 53.2 and 45.5% using 0.05, 0.10, 0.15 and 0.20 M sodium hydroxide solution respectively using seventh cycle. Using 0.05, 0.10, 0.15 and 0.20 M potassium hydroxide solution, these efficiencies were achieved by 77.5, 87.5, 71.2 and 65.5% after first cycle respectively; and decreased by 41.2, 44.2, 39.5 and 35.4% after seventh cycle respectively. Further, desorption efficiency of Congo red dye from GLP were achieved to 65.2–25.5% using 0.05 to 0.20 M lithium hydroxide, calcium hydroxide and sodium bicarbonate solution as an eluent in first cycle.

**Table 2:** Results of batch desorption study of Crystal Violet dye and Carbonate ion.

Eluent	Nos. of cycles	Desorption efficiency (%)	
		Congo Red dye	Carbonate ion
0.05 to 0.20 M acid solution (HCl, H_2_SO_4_, HNO_3_, CH_3_COOH, EDTA)	First	1.0–2.3	1.2–2.8
Solvents (rectified spirit, acetone, acetaldehyde, toluene and DCM)	First	10.7–15.1	12.2–14.5
0.05, 0.10, 0.15 and 0.20 M NaOH solution	One	87.5, 98.5, 80.2 and 74.5	86.5, 96.2, 84.4 and 70.2
	Eight	50.4, 58.4, 53.2 and 45.5	50.2, 55.6, 53.7 and 48.7
0.05, 0.10, 0.15 and 0.20 M KOH solution	One	77.5, 87.5, 71.2 and 65.5	90.4, 94.5, 75.5 and 60.5
	Eight	41.2, 44.2, 39.5 and 35.4	35.5, 39.5, 36.4 and 30.2%
0.05 to 0.20 M LiOH solution	First	65.2–45.5	62.2–41.2
0.05 to 0.20 M Ca(OH)_2_ solution	First	55.4–40.2	50.2–35.2
0.05 to 0.20 M NaHCO_3_ solution	First	35.7–25.5	33.5–23.5

Same elution behaviour of carbonate ion from exhausted NLP was observed. Strong as well as weak acid shows the 1.2–2.8% desorption efficiency. Also, desorption efficiencies were attained up to 12.2–14.5% using all the solvents (rectified spirit, acetone, acetaldehyde, toluene and DCM) as an eluent after first cycles. Using 0.05, 0.10, 0.15 and 0.20 M sodium hydroxide solution, higher desorption efficiencies i.e. 86.5, 96.2, 84.4 and 70.2% respectively after first cycle; and 50.2, 55.6, 53.7 and 48.7% respectively after seven cycle were obtained. Also, desorption efficiencies were reached to 90.4, 94.5, 75.5 and 60.5% after first using 0.05, 0.10, 0.15 and 0.20 M potassium hydroxide solution respectively, which was decreased to 35.5, 39.5, 36.4 and 30.2% respectively after seventh cycle. Further, efficiencies were attained from 65.2 to 23.5% using 0.05 to 0.20 M lithium hydroxide, calcium hydroxide and sodium bicarbonate solution as an eluent in first cycle.

From above results, it is reveal that higher desorption efficiencies of anionic Congo red dye or carbonate ion from adsorbent, GLP or NLP respectively using alkaline solution as an eluent, while solvents and acidic solutions were desorbed lesser amount of Congo red dye or carbonate ion. This behaviour attributed to the fact that anionic acid Congo red or carbonate ion were adsorbed onto cationic adsorbent, GLP or NLP respectively. As quantity of adsorbent is abundant higher than adsorbate at equilibrium, so, exhausted adsorbent, GLP or NLP became cationic. Now, cationic adsorbent was reacted with negative charged different alkali, so, negative charge adsorbate i.e. anionic Congo red dye or carbonate ion was eluted from exhausted GLP or NLP due to electrostatic interaction. Further, strong concentration of alkaline solution (0.15 and 0.20 M NaOH) may be damage the structure of exhausted adsorbent and lower concentration of alkaline solution (0.05 M NaOH and all solutions of KOH, LiOH, Ca(OH)_2_ and NaHCO_3_) may be slower he desorption mechanism. Solvents, having different functional groups, has slightly affinity to of anionic Congo red dye or carbonate ion, so, minor desorption occurs. But cationic exhausted adsorbent, GLP or NLP was not or negligible eluted anionic Congo red dye or carbonate ion using cationic acidic solution due to common ion effect. Hence, 0.1 M NaOH shows highest desorption capacity for anionic Congo red dye and carbonate ion.

Table 3 depicted desorption efficiency of Methylene blue, Cadmium (Cd) Cadmium (Cd) from NLP, NLP and charcoal prepared from NLP respectively using acids, alkali and solvents as an eluent, in which acidic solutions show excellent results, while alkaline solutions and solvents had least results. Using 0.05, 0.10, 0.15 and 0.20 M HCl solution, desorption efficiencies of methylene blue dye from NLP were obtained 93.5, 97.8, 81.2 and 75.5% respectively after first cycle. Then, continuous decrements were found after completion of each cycle. Finally, these efficiencies were decreased up to 55.5, 60.2, 50.7 and 48.5% using 0.05, 0.10, 0.15 and 0.20 M HCl solution respectively after seventh cycle. The desorption efficiencies were attained to 87.5, 91.5, 80.3 and 70.5% after first cycle and 53.9, 57.5, 50.2 and 42.5% after seventh cycle using 0.05, 0.10, 0.15 and 0.20 M H_2_SO_4_ solution respectively. Moreover, desorption efficiency of methylene blue dye from NLP was achieved in range of 53.2 to 30.2% using 0.05 to 0.2 M HNO_3_, CH_3_COOH and EDTA solution. Also, least efficiencies i.e. 11.5–13.2% and 0.9–2.1% were observed using different solvents and alkaline solutions respectively.

**Table 3:** Results of batch desorption study of Methylene blue and Cadmium (Cd).

Eluent	Nos. of cycles	Desorption efficiency (%)		
		Methylene Blue dye	Cadmium (Cd) from NLP	Cadmium (Cd) from activated char from NLP
0.05, 0.10, 0.15 and 0.20 M HCl solution	One	93.5, 97.8, 81.2 and 75.5	90.1, 93.4 and 81.1 70.2	95.4. 97.2, 80.5 and 78.8
	Seventh	55.5, 60.2, 50.7 and 48.5	51.2, 55.7 and 50.2 44.4	54.9, 59.8, 55.4 and 47.5
0.05, 0.10, 0.15 and 0.20 M H_2_SO_4_ solution	One	87.5, 91.5, 80.3 and 70.5	80.7, 84.5, 74.2 and 65.4	86.5, 89.7, 80.5 and 70.2
	Seventh	53.9, 57.5, 50.2 and 42.5	45.1, 50.2. 45.8 and 39.5	55.2, 57.8 and 50.4 41.9
0.05 to 0.20 M HNO_3_ solution	First	53.2–40.2	48.5–38.8	52.9–43.0
0.05 to 0.20 M CH_3_COOH solution	First	45.3–35.9	39.5–30.6	44.7–32.6
0.05 to 0.20 M EDTA solution	First	39.9–30.2	34.5–27.5	38.2–31.2
Solvents (rectified spirit, acetone, acetaldehyde, toluene and DCM)	First	11.5–13.2	11.8–14.1	12.5–15.6
0.05 to 0.20 M alkali (NaOH, KOH, LiOH, Ca(OH)_2_ and NaHCO_3_)	First	0.9–2.1	1.0–2.6	1.2–3.0

The desorption efficiencies of Cadmium (Cd) from NLP were reached in range of 93.4–70.2% after first cycle and 55.7–44.4% after seventh cycle using 0.05–0.20 M HCl solution respectively, while using other acidic solutions (0.05–0.2 M HNO_3_, CH_3_COOH and EDTA solutions), different solvents and alkaline solution, lower efficiencies were attained in range of 48.5–27.5, 11.8–14.1 and 1.0–2.6% respectively. Further, Cadmium desorption from AC-NLP was performed and found that efficiencies were achieved up to 97.2–78.8% after first cycle and 59.8–47.5% after seventh cycle using 0.05–0.20 M HCl solution. Also, these efficiencies were attained up to 89.7–31.2, 12.5–15.6 and 1.2–3.0% using other acidic solutions (0.05–0.20 M HNO_3_, CH_3_COOH and EDTA solutions), different solvents and alkaline solution respectively.

Here, acidic solution as an eluent shows greater desorption efficiencies for elution of cationic methylene blue dye and cadmium (Cd) metal from NLP and AC-NLP. But, solvents and alkali solutions show least desorption behavior. Here, first cationic methylene blue dye and cadmium (Cd) were adsorbed onto anionic NLP and AC-NLP. Amount of adsorbent is plentiful higher than adsorbate at equilibrium, so, positive charged (anionic) was occurs on exhausted NLP and AC-NLP. Then, anionic exhausted was contacted with positive charged acidic solution, which eluted cationic methylene blue dye and cadmium (Cd) metal from NLP and AC-NLP due to electrostatic interaction. Further, strong acid (H_2_SO_4_) and weak acids (HNO_3_, CH_3_COOH and EDTA) shows lesser desorption than using moderate potential acid (HCl). Higher concentration i.e. 0.15 and 0.20 M HCl may be damage the structure of adsorbent and lower concentration i.e. 0.05 M HCl may be delay the desorption process. While, solvents and alkali solution shows slight and negligible desorption. Also, it was observed that AC-NLP has higher desorption efficiency for cadmium than NLP. Thus, 0.1 M HCl shows maximum desorption capacity for methylene blue dye and Cadmium (Cd) metal. Further, activated charcoal has three types of pores according to diameters i.e. micropore, mesopore and macropore, so, eluent may be easily penetrated into these pores of AC-NLP than pure NLP.

From this section, we concluded that higher desorption efficiencies of anionic Congo red dye or carbonate ion from adsorbent, GLP or NLP respectively using acid as an eluent. Also, alkaline solution as an eluent shows greater desorption efficiencies for elution of cationic methylene blue dye and cadmium (Cd) metal from NLP and AC-NLP. Strong acid may be destroyed the structure of adsorbent and weak acid may be slower the desorption mechanism. AC-NLP has higher desorption efficiency for cadmium than native NLP. Hence forward, anionic and cationic adsorbate will be eluted using 0.1 M hydrochloric acid and 0.1 M sodium hydroxide solution respectively in kinetic and column study^8^.

To determine the desorption mechanism, elution profile of Congo red dye and cadmium metal was studied using 0.1 M hydrochloric acid and 0.1 M sodium hydroxide solution respectively for contact duration of 30, 60, 120, 180, 240 and 300 min. These data were evaluated by kinetic isotherm i.e. Pseudo-First order, Pseudo-Second order, Intra-particle diffusion and Elovich isotherm. Table 4 represented the enlisted kinetic isotherms used, their equations, their linear plots, and their correlation coefficient values (R^2^) for elution of Congo red and cadmium. Highest R^2^ values (0.9987 and 0.9974) were obtained from Pseudo-First order. Another kinetics isotherm had R^2^ values were in range of 0.9900–0.9711. These values revealed that desorption of Congo red and cadmium follows Pseudo-First order. This is due the fact that desorption process depends upon two reactants i.e. adsorbate and eluent, but quantity of eluent is too much excess compared to adsorbate. So, rate of desorption is depends upon adsorbate only.

**Table 4:** Desorption kinetic data.

Kinetic model	Equation	Plot	Correlation Coefficient (R^2^)	
			Congo Red dye	Cadmium metal
Pseudo-first order	ln(q_e_ − q_t_) = ln q_e_ − k_1_t	In(q_t_ − q_e_) vs t	0.9984	0.9974
Pseudo-second order	t/q_t_ = (1/k_2_q_e_^2^) + (1/q_e_)t	t/q_t_ vs t	0.9711	0.9778
Intra-particle diffusion	q_t_ = k_p_ t^1/2^ + C	q_t_ vs. t^1/2^	0.9800	0.9745
Elovich isotherm	q_t_ = 1/β In (αβ) + 1/β In t	q_t_ vs. ln t	0.9889	0.9900

In Where, q_t_ and q_e_ are the amounts of Congo red or cadmium desorbed at time t and equilibrium (mg/g) respectively, t is time in minutes, k_1_, k_2_ and k_p_ are the Pseudo first-, Second- order and Intra-Particle diffusion rate constant respectively, α and β are Elovich constant.

### Column study

Desorptive column study was performed using 0.1 M hydrochloric acid and 0.1 M sodium hydroxide solution for elution of Congo red and cadmium metal from GLP and AC-NLP respectively at flow rate 5 mL/min and bed height of 10 cm; total seven cycles were performed. From these data, breakthrough and desorption curves were plotted. First, sorption column was packed with exhausted adsorbent i.e. GLP or AC-NLP (quantity: 12.4–10.5 g) with packing density 215 g/L and bed height of 10 cm. And after completion of seven cycle, only 9.5–7.4 g dry adsorbent mass having packing density 151.1–149.2 g/L and bed height of 8.7–8.0 cm was left behind in column, which shows weight loss of 30.1–35.0%.

**Figure 1:**
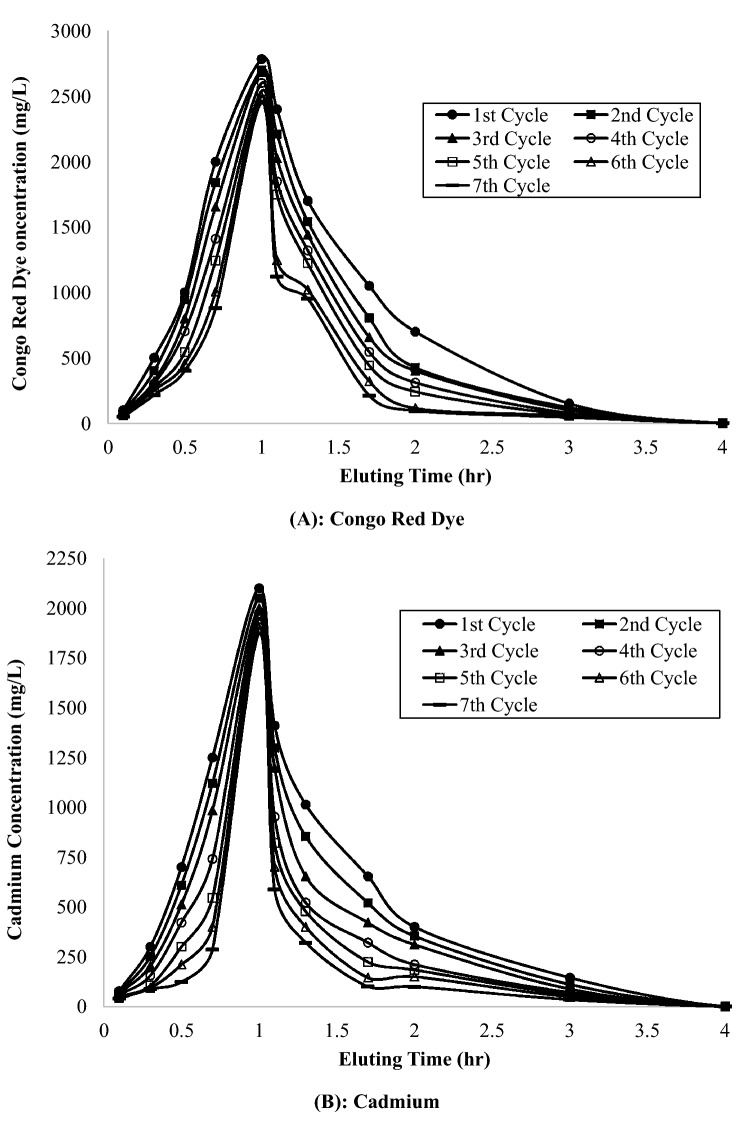
Elution breakthought curves of Congo Red Dye and Cadmium metal.

The flow rate of desorption (5 mL/min) is selected slightly lower than previous adsorption study (10 mL/min), which promotions higher sorption of the sorbate into the available sites on the adsorbate resulting in a higher binding^17^. Figure 1 shows the elution breakthrough curves of Congo red dye and cadmium metal, which shows higher elution of adsorbate from NLP and AC-NLP initially. Thereafter, slow elution of both adsorbates was presented. Same type of desorption behaviour were observed by Fagundes-Klen et al.^18^ and Lodeiro et al.^19^ in their experiments. Also, Table 5 depicted the different sorption and elution process parameters for seven sorption–desorption cycles. The desorption process slowly decreased with increment of number of cycles, as per batch study. The percentage update capacity of dye was obtained by 70.0 to 64.8 mg/g for dye and 25.0 to 22.0% for metal from first cycle to seventh cycle respectively. Further, percentage removal of dye (85.5 to 56.3%) and metal (88.8 to 60.5%) was decreasing with number of cycles (Table 5).

**Table 5:** Sorption and elution process parameters for seven sorption–desorption cycles.

Adsorbate	Cycle no	Update capacity (mg/g)	t_b_ (min)	t_e_ (min)	∆t (min)	dC/dt (mg/l h)	Z (cm)	Z_m_ (cm)	Percent removal (%)	Elution efficiency (%)
Congo Red dye	1	70.0	42.5	65	22.5	8.5	10.0	6.3	85.5	99.4
	2	68.5	34.8	60	25.2	7.9	9.9	6.4	78.8	99.1
	3	67.9	29.9	57.4	27.5	7.1	9.6	6.4	72.5	98.8
	4	67.0	22.5	55.1	32.6	5.7	9.0	6.6	67.9	98.8
	5	66.6	17.4	52.6	35.2	5.6	8.7	6.9	62.5	98.4
	6	65.2	9.5	47.6	38.1	4.5	8.4	6.9	58.5	97.8
	7	64.8	1.1	40	38.9	3.3	8.0	7.2	56.3	97.5
Cadmium metal	1	25.0	44.4	67.8	23.4	8.1	10.0	6.4	88.8	99.7
	2	24.8	32.4	62.7	30.3	7.8	9.8	6.4	86.5	99.3
	3	24.2	26.5	57.6	31.1	7.1	9.6	6.6	82.2	98.5
	4	23.7	21.5	55.1	33.6	6.5	9.6	6.6	78.5	98.0
	5	23.1	17.6	52.9	35.3	4.2	9.0	6.8	72.2	97.8
	6	22.4	12.6	50.1	37.5	3.8	8.9	7.0	68.8	97.3
	7	22.0	7.5	45.8	38.3	2.9	8.7	7.2	60.5	97.0

**Figure 2:**
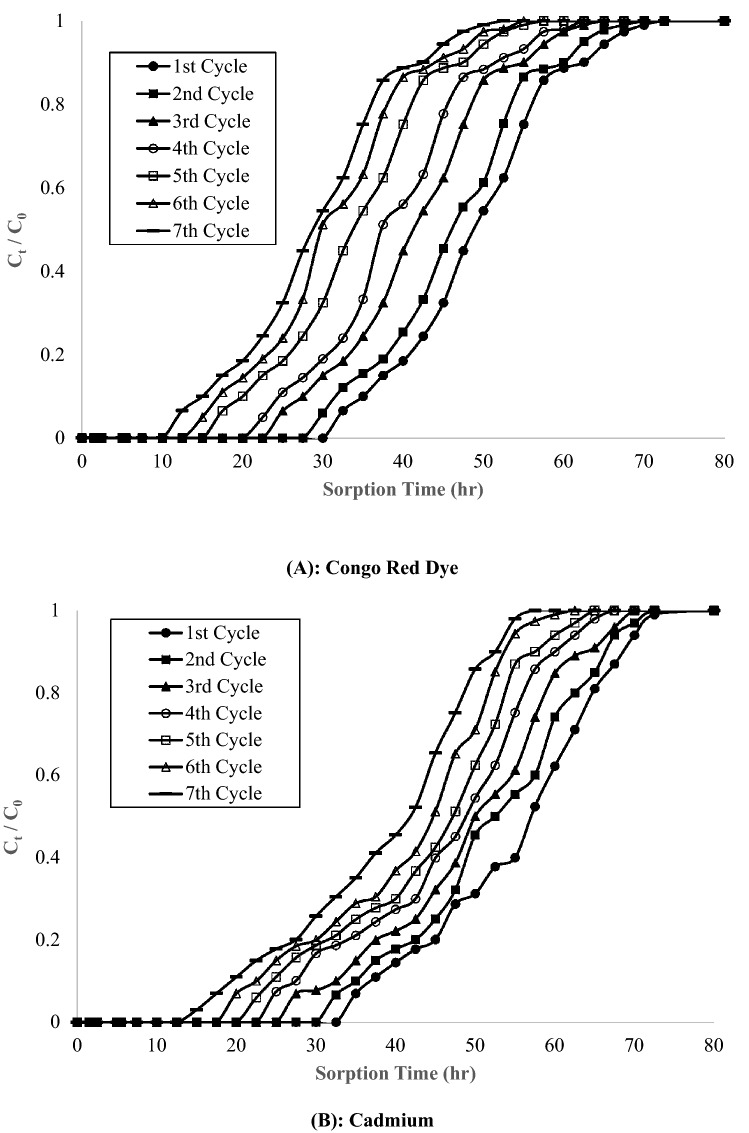
Sorption breakthought curves of Congo Red Dye and Cadmium metal.

Figure 2 depicted the sorption breakthrough curves for Congo red and cadmium. Both the adsorbates had lowest slope initially, which revealed high adsorption capacity in the first cycle. Then, with increasing number of cycles, the slope became sleeper, which show lower adsorption capacity. It is also confirmed by decreasing value of rate constant-dC/dt (8.5 to 3.3 mg/L·h for Congo red and 8.1 to 2.9 mg/L·h for Cadmium) and enlargement of mass transfer zone-∆t (22.5 to 38.9 cm for Congo red and 23.4 to 38.3 cm for Cadmium) with number of cycles, mentioned in Table 5. Further, equilibrium was attained after 3.6 h for both adsorbates, which lower than batch desorption study (6.0 h). Also, total quantity of eluent used for batch and column study were 250 mL and 189.5 mL per exhausted adsorbent respectively. So, it is revealed that column desorption study is more feasible than batch study.

**Figure 3:**
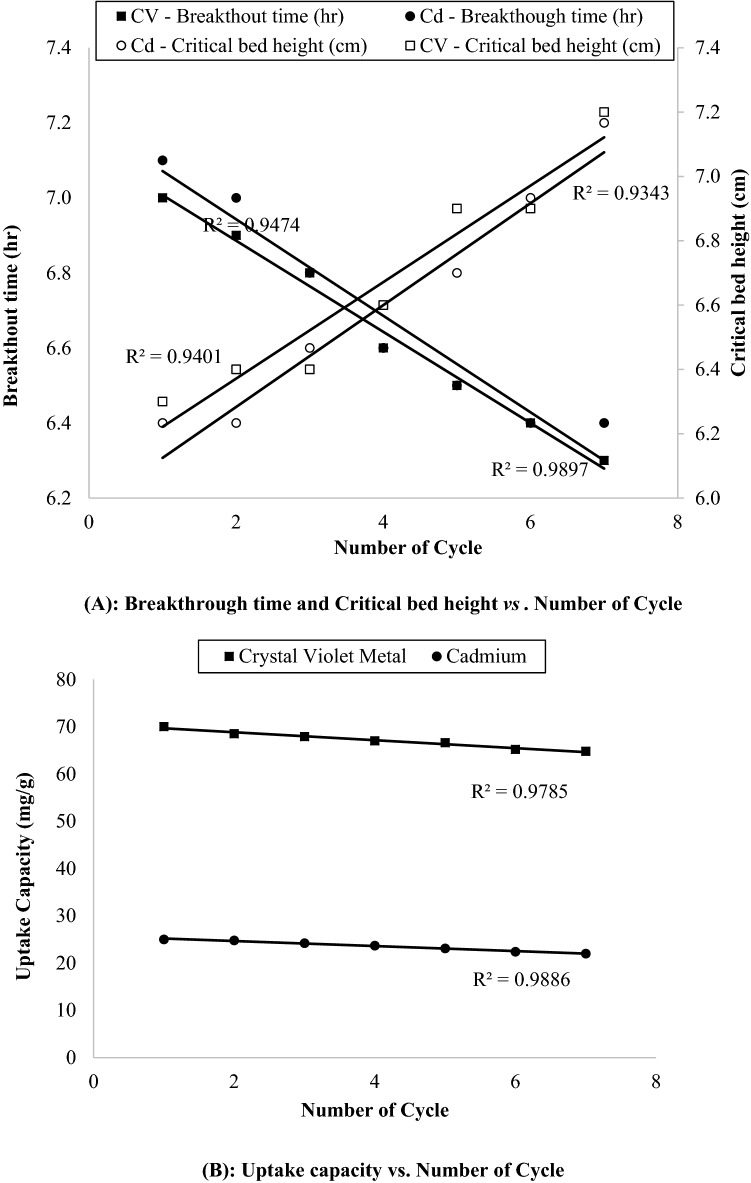
Linear plots of breakthrough time, critical bed height and Uptake capacity versus number of cycles.

Figure 3A,B represents the breakthrough time and critical bed length vs*.* number of cycle and uptake capacity vs. number of cycle (*x*) respectively, which is straight line having coefficient of determination (R^2^) value greater than 0.93. This indicate the applicability of all activity-indicator equation in desorption study. Table 6 presents the different parameter of activity-indicator equation. From the plot of breakthrough time vs. number of cycle i.e. *x*, t_b,0_ and k_tb_ were attained up to 7.1286 h and 0.1286 h/cycle for Congo red respectively; and 7.2 h and 0.1214 h/cycle for cadmium respectively. Therefore, it can be assisted that sufficient capacities to escape the breakthrough at time t = 0 for up to 55 cycles for both adsorbates, Congo red and cadmium. Value of Q_0_ and k_Q_ were achieved up to 70.5 mg/g and 0.8393 mg/g·cycle for Congo red respectively; and 24.729 mg/g and 0.5321 mg/g·cycle respectively derived from plot of uptake capacity vs.* x*. Thus, it can anticipated that column may be completely exhausted i.e. no uptake after 84 and 48 cycles for Congo red and cadmium respectively. These two different values implies due to the fact that initial concentration of methylene blue and cadmium were different i.e. 75 and 25 mg/g respectively in previous adsorption experiments. Also, from the plot of critical bed length vs.* x*, Z_m,0_ and kZ_m_ were attained up to 9.0174 cm and 0.15 cm/cycle for Congo red respectively; and 6.1714 cm and 0.1357 cm/cycle for cadmium respectively, which shows that breakthrough would be performed after 40–45 cycles at time t = 0.

**Table 6:** Parameter of activity-indicator equation.

Plot	Parameters	Congo Red dye	Cadmium metal
Breakthrough time (t_b_) vs. Number of cycle (*x*)	t_b,0_ (h)	7.1286	7.20
	k_tb_ (h/cycle)	0.1286	0.1214
	R^2^	0.9897	0.9494
Uptake capacity (Q) vs. Number of cycle (*x*)	Q_0_ (mg/g)	70.5	24.729
	k_Q_ (mg/g·cycle)	0.8393	0.5321
	R^2^	0.9785	0.9986
Critical bed height (Z_m_) vs. Number of cycle (*x*)	Z_m,0_ (cm)	9.0714	6.1714
	kZ_m_ (cm/cycle)	0.1500	0.1357
	R^2^	0.9343	0.9401

## Conclusion

1. Feasibility of elution of anionic and cationic compounds from previously exhausted adsorbents using different eluents was analysed in this paper.
2. Batch desorption study was performed for elution of dye, metal and ions from exhausted adsorbents using five acidic, alkaline and five solvents. Consecutive seven cycles were conducted, and desorption efficiencies were determined.
3. It shows the higher desorption efficiency of anionic (97.8%) and cationic (98.5%) adsorbates using moderate concentration of acidic and alkaline solution respectively due to electrostatic interaction, while solvent shows less elution. AC-NLP (97.2%) has higher desorption efficiency for cadmium than native NLP (93.4%). Pseudo-First order is most fitted among used models, derived from correlation coefficient.
4. Column elution of Congo red dye and Cadmium metal as adsorbate using 0.1 M hydrochloric acid and 0.1 M sodium hydroxide solution as an eluent respectively; and breakthrough and desorption curves were plotted. It revealed that initial higher elution and after sometimes, slow elution of adsorbate was observed from elution breakthrough curves. Also, sorption breakthrough curves concluded that adsorption efficiencies were decreasing i.e. 99.7 to 97.5% with increasing the number of cycle i.e. one to seven cycles respectively.
5. Sorption and elution process parameters for seven sorption–desorption cycles were calculated and mentioned. Plots for activity-indicator equations were drawn and their parameters were tabulated, which indicates that column may be completely exhausted after 84 and 48 cycles for Congo red and cadmium respectively.
6. Column desorption is more feasible than batch desorption study with respect to desorption equilibrium time and quantity of eluent per exhausted adsorbent.
